# Quantity and Quality of Aquaculture Enrichments Influence Disease Epidemics and Provide Ecological Alternatives to Antibiotics

**DOI:** 10.3390/antibiotics10030335

**Published:** 2021-03-22

**Authors:** Anssi Karvonen, Ville Räihä, Ines Klemme, Roghaieh Ashrafi, Pekka Hyvärinen, Lotta-Riina Sundberg

**Affiliations:** 1 Department of Biological and Environmental Science, University of Jyvaskyla, P.O. Box 35, 40014 Jyvaskyla, Finland; ville.p.raiha@jyu.fi (V.R.); ines.klemme@jyu.fi (I.K.); roghaieh.ashrafi@jyu.fi (R.A.); lotta-riina.sundberg@jyu.fi (L.-R.S.); 2 Natural Resources and Bioproduction, Natural Resources Institute Finland (Luke), Manamansalontie 90, 88300 Paltamo, Finland; pekka.hyvarinen@luke.fi; 3 Nanoscience Center, University of Jyvaskyla, P.O. Box 35, 40014 Jyvaskyla, Finland

**Keywords:** aquaculture, biofilm, disease epidemiology, enriched rearing, environmental microbes, microbial community, *Salmo salar*, *Salmo trutta*

## Abstract

Environmental heterogeneity is a central component influencing the virulence and epidemiology of infectious diseases. The number and distribution of susceptible hosts determines disease transmission opportunities, shifting the epidemiological threshold between the spread and fadeout of a disease. Similarly, the presence and diversity of other hosts, pathogens and environmental microbes, may inhibit or accelerate an epidemic. This has important applied implications in farming environments, where high numbers of susceptible hosts are maintained in conditions of minimal environmental heterogeneity. We investigated how the quantity and quality of aquaculture enrichments (few vs. many stones; clean stones vs. stones conditioned in lake water) influenced the severity of infection of a pathogenic bacterium, *Flavobacterium columnare*, in salmonid fishes. We found that the conditioning of the stones significantly increased host survival in rearing tanks with few stones. A similar effect of increased host survival was also observed with a higher number of unconditioned stones. These results suggest that a simple increase in the heterogeneity of aquaculture environment can significantly reduce the impact of diseases, most likely operating through a reduction in pathogen transmission (stone quantity) and the formation of beneficial microbial communities (stone quality). This supports enriched rearing as an ecological and economic way to prevent bacterial infections with the minimal use of antimicrobials.

## 1. Introduction

Infectious diseases represent one of the greatest threats for intensive food production. For example, a significant proportion of annual crop yield are lost because of diseases [1,2]. Similarly, diseases of farmed animals, such as those occurring in aquaculture, are causing losses summing up to several billion USD annually [3,4]. Disease-related problems are also expected to exacerbate with the ongoing climate change [4,5,6,7], which can be predicted to result in increasing use of chemicals in disease treatment and eradication. The use of chemicals and medication generate further economic costs. In addition, treatments often become ineffective as pathogens evolve resistance to antimicrobials and can result in other problems such as environmental pollution [8,9]. Thus, ecological and economic means to prevent and fight infections in intensive farming environments, such as new innovative methods and modifications of conventional farming practices, are currently being sought.

One of the key factors influencing the epidemiology of disease-causing agents in natural and farming systems is the heterogeneity or structure of the environment [10,11,12,13,14,15]. The rate of disease transmission is determined by contacts between infected and susceptible hosts, with the structure of the environment influencing their distribution and consequently, the spread and persistence of a disease [16]. Environmental heterogeneity can also refer to the presence or diversity of other hosts, pathogens and environmental microbes, which can dilute exposure [17,18], interact with the primary infection [19,20,21,22] and limit the occurrence and epidemics of pathogens [23,24]. For instance, the importance of microbial communities inside and outside a host for infections, immune function and host health is becoming increasingly evident [25,26], with examples including diverse taxa from mammals and birds [27,28] to amphibians and fish [29,30,31,32,33].

Environmental heterogeneity can also have direct epidemiological relevance for diseases occurring in farming environments, such as aquaculture. Often these systems are characterized by unnaturally high host densities (dense monocultures of one species), minimal environmental heterogeneity (simplified rearing facilities), high level of hygiene (removal of biofilms) and use of disease preventatives (chemicals, antimicrobials, vaccines). High host densities, together with minimized influence from the surrounding environment, create favourable epidemiological conditions for disease transmission between fish. However, recent studies have begun to investigate the role of increased environmental heterogeneity, i.e., the enrichment of the rearing environment, in preventing aquaculture diseases. These studies have shown that enrichments can significantly reduce the mortality of fish associated with bacterial and protozoan infections, despite some degree of variation in these effects depending on the species-specific host–pathogen combinations [34,35]. However, there is still very little knowledge on the effects of quantity and quality of enrichments on the occurrence and severity of fish diseases. This information is important in elucidating the detailed mechanisms underlying the effects of enriched rearing and designing the most effective rearing methods to minimise the use of antimicrobials.

In the present study, we investigated the influence of the quantity and quality of environmental enrichments (natural stones) on the epidemiology of the bacterium *Flavobacterium columnare* in aquaculture conditions. *Flavobacterium columnare* is transmitted directly between fish and is currently considered as one of the most harmful pathogens in aquaculture. The infection causes epidermal lesions on fish [36], which can result in significant morbidity and mortality [37,38]. Infections are treated with antibiotics, which has resulted in some bacterial strains becoming at least partially resistant to treatments. Furthermore, minor antibiotic concentrations spilling over into the environment can modify interactions between different bacterial species [39,40] and other, non-targeted parasites [41], thus extending the effects of antimicrobial usage outside the aquaculture realm.

We experimentally exposed two important aquaculture fish species, sea-migrating brown trout (*Salmo trutta*) and landlocked Atlantic salmon (*Salmo salar* m. *sebago*), to the bacterium and manipulated the quantity of stones (no stones, few or many) and their quality (clean unconditioned stones, or conditioned stones that had been maintained in lake water) in the exposure tanks. We predicted that a higher number of stones would facilitate their use as shelters, thus decreasing contacts between the fish, and resulting in lower bacterial transmission and mortality among fish. We also expected that the prior conditioning of the stones in aquaculture tanks could inhibit the spread of the disease and associated mortality of fish, for example, if the stones housed beneficial microbial communities operating against *F. columnare*.

## 2. Results

**Figure 1:**
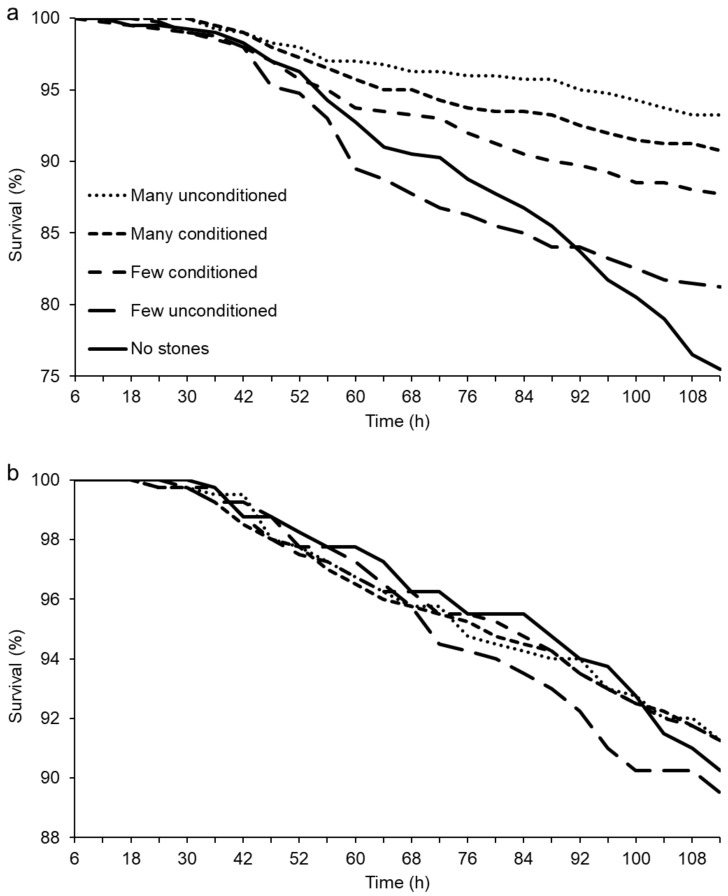
Survival of trout and salmon in experimental bacterial exposure. Cumulative survival curves of **a**) sea-migrating brown trout (*Salmo trutta*) and **b**) landlocked Atlantic salmon (*Salmo salar* m. *sebago*) exposed to the pathogenic bacterium *Flavobacterium columnare*. Exposures of groups of 100 fish took place in 50 replicated tanks (0.4 m^2^) with different combinations of quantity (no/few (6 stones)/many (60 stones)) and quality (unconditioned/conditioned) of natural stones. Conditioning was done by keeping clean stones in the lake water before the experiment. Survival was followed every 4–6 h until 112 h post-exposure. In trout, survival was lowest in tanks without stones, but increased in the presence of conditioned stones as well as with an increasing number of unconditioned stones (**a**). Similar trends of lowest survival in tanks without stones and with few unconditioned stones were evident also in salmon at the end of the experiment (**b**). Note differences in Y axis scales.

The survival of trout was affected by an interaction between the quantity (few/many) and quality (unconditioned/conditioned) of the stones (Table 1, Figure 1a). Pairwise comparisons indicated that conditioning improved the survival of trout when stones were few (generalized linear mixed model (GLMM): χ^2^ = 5.081, *p* = 0.024), but this effect was no longer significant when stones were many (χ^2^ = 1.768, *p* = 0.184). Additionally, a higher number of unconditioned stones improved the survival of trout compared to tanks with few unconditioned stones (χ^2^ = 22.076, *p* < 0.001), but no such effect of stone numbers was observed with conditioned stones (χ^2^ = 0.844, *p* = 0.358). Furthermore, tanks with few conditioned stones showed a significantly higher survival of trout compared to tanks without stones (χ^2^ = 10.114, *p* = 0.001), while the higher survival in tanks with few unconditioned stones compared to tanks without stones was not significant at the five percent level (χ^2^ = 2.492, *p* = 0.114).

In salmon, the patterns of survival among the treatments were somewhat similar to trout. The “no stones” and “few unconditioned stones” treatments showed the lowest survival (48.7% of deceased fish were recorded in those two treatment groups) and the differences became increasingly evident during the last 24 h of the experiment (Figure 1b). However, due to the lower overall mortality in salmon (8.7%) compared to trout (13.9%), and the high within-treatment variation in mortality between replicate tanks, survival differences in salmon were not statistically significant (GLMM: *p* > 0.05 for all). A total of seven trout (1.4%) and seven salmon (1.4%) died in the control tanks.

## 3. Discussion

Environmental heterogeneity can significantly influence the epidemiology of infectious diseases [10,11,12]. However, intensive farming units are typically characterized by very little or no environmental structuring. While recent research suggests positive health effects associated with the enrichment of aquaculture environments, the effects of type and number of enrichments on the occurrence and severity of diseases are not well understood. Such information is important for questions of when and how enrichments should be applied most effectively to minimise the use of antimicrobials. We investigated how the quantity and quality of environmental enrichments (stones) influenced the epidemiology of *Flavobacterium columnare* infecting its aquaculture fish hosts. The conditioning of the stones in aquaculture tanks significantly reduced the mortality of trout among tanks with few stones. Furthermore, increasing the number of unconditioned stones from few to many also improved fish survival to the level corresponding to that with conditioned stones.

The enrichment of the aquaculture environment has been shown to have positive effects on several life-history traits of fish [42,43,44,45,46,47,48,49]. Experimental studies have also supported the role of enrichments in decreasing the disease-related mortality of fishes [34,35], but these earlier studies did not manipulate the quantity or quality of the enrichments. Here, our results indicate that just a handful of stones can significantly influence the bacterial epidemic, particularly if the stones have been previously conditioned in aquaculture tanks. Although not analysed in detail, the most plausible explanation for the higher fish survival is that the conditioned stones harboured microbial communities that inhibited the *F. columnare* epidemic. Recent studies have begun to investigate the role of environmental microbes of biofilms in suppressing the occurrence of pathogens [23,24] and *F. columnare* has been shown to be a poor competitor against other bacterial species [50,51,52]. Regardless of the underlying mechanism, such a strong effect on disease epidemiology owing to just six stones is surprising and suggests that even a minimal environmental enrichment can improve the health of aquaculture fish. This is further emphasized by the fact that this beneficial effect was not amplified in tanks with a higher number of conditioned stones. Thus, conditioned enrichments seem to have a threshold effect related to their presence or absence. Studies are needed on factors such as the size and shape of the stones related to their surface area, and the duration of the conditioning and rate of water change in the tanks, influencing the possible establishment and composition of microbial communities.

We also observed similar higher fish survival with the higher number of unconditioned stones. This finding may be related to the epidemiological progression of *F. columnare* infection. Visual observations from the tanks with many stones suggested that most fish were solitary and resided next to the stones, in contrast to the tanks with no stones or few stones, where fish tended to form groups (these behaviours were not quantified in detail). While all fish were probably exposed to the bacterium in the beginning of the experiment, they may subsequently release the bacterium at different rates, depending on the state of the infection. For example, the shedding of *F. columnare* by diseased fish increases after the initial exposure and is particularly high in deceased fish due to the saprophytic nature of the bacterium [53]. Thus, it is possible that the increasing number of stones reduced contacts between fish shedding the bacterium (moribund or deceased fish) and those appearing healthy. However, detailed mechanisms underlying the epidemiological progression of *F. columnare* infection in a tank, such as the role of moribund fish in fuelling the epidemic, are currently unknown.

It is also possible that the higher survival of fish in tanks with many stones is related to their higher resistance if, for example, occupying individual habitats next to the stones decreased stress. Stress has often been related to disease resistance in fish [54,55,56] and generally in many other organisms [57,58,59,60]. However, no significant effect of stone numbers on fish survival was observed with conditioned stones, which does not support the idea of reduced stress. Furthermore, in an earlier study with moderate production-scale fish densities of 1250 fish per tank, we observed a strong beneficial effect of stone enrichments against *F. columnare* [35]. However, the number of stones per fish in those tanks roughly corresponded to that in the few stones treatment in the present study. This means that, in practice, most of the fish could not be residing next to stones at the same time. In Räihä et al. (2019) [35], the natural bacterial epidemic also occurred several weeks after the enrichments had been introduced to the tanks, which is why the stones likely housed well-established microbial communities similarly to the conditioned stones in the present investigation. Thus, the results in Räihä et al. (2019) [35] could also indirectly support the effect of stone quality rather than their quantity.

We did not detect the significant effects of tank enrichment in salmon although, similarly to trout, the survival of these fish in tanks without stones and a few unconditioned stones tended to decrease towards the end of the experiment. In general, trout is known to be more susceptible to *F. columnare* than salmon, with even a 20-fold difference in mortality during a natural epidemic [35]. Furthermore, the epidemiological progression of the disease is slower in salmon, with mortality associated with *F. columnare* increasing only after 100 h post-exposure in experimental conditions [61]. As the duration of the present experiment was relatively short (112 h) due to technical reasons, it is possible that marked differences in the patterns of mortality between the treatments could have emerged later, following the trajectories already evident here at 100–112 h post-exposure. Thus, the results on salmon should be interpreted with caution.

To conclude, we found that even a simple environmental enrichment can reduce the effect of diseases in aquaculture environment. This may have important implications for questions of when and how aquaculture enrichments should be applied. For example, if a constant maintenance of enrichments in tanks increased labour costs or complicated the cleaning of tanks, it could be possible to apply previously conditioned enrichments just before an anticipated disease outbreak. Our results indicate that such a short-term enrichment of the exposure environment with just a few conditioned enrichments (in this case stones) can already have a significant positive effect on fish survival, which could reduce the need for a medical intervention. However, issues such as the timing and duration of enrichment in relation to the occurrence of diseases, and the number and type of enrichments needed in relation to the number or density of fish, would need to be considered individually for each aquaculture facility. Overall, we suggest that even a simple form of enriched rearing, when tailored to the specific conditions and needs of a rearing facility, could provide an effective means to reduce disease incidence and the use of antimicrobials in aquaculture.

## 4. Materials and Methods

A controlled exposure experiment was designed to study the effects of the quantity and quality of natural enrichment (stones) on the mortality of sea migrating brown trout (*Salmo trutta*) and landlocked Atlantic salmon (*Salmo salar* m. *sebago*) (hereafter “trout’” and “salmon”) caused by the bacterium *F. columnare*. In the experiment, the fish were exposed to controlled doses of the bacterium in tanks with no stones, or with stones of different quantity (few/many) and quality (unconditioned/conditioned). The experiment was conducted at Kainuu Fisheries Research Station (KFRS) of the Natural Resources Institute Finland in Paltamo (www.kfrs.fi (accessed on 15 December 2020), 64.404°N, 27.516°E) in July 2018. The station is a flow-through facility, taking water from a nearby Lake Kivesjärvi (from the depth of 7 m). Water temperature in the facility follows the natural temperature of the lake and all experimental units receive the same water supply.

Trout and salmon originated from the parent fish whose numbers varied between 60 and 69 per species, and 4–5 females each crossed with 3–4 males. The fish were divided in 3.2 m^2^ tanks at the eye-egg stage, using 2 replicate tanks with 3000 fish each for each species, in total 4 tanks. As the fish grew larger, they were divided into 4 tanks per species with 1500 fish in each, in total 8 tanks. All tanks were equipped with environmental enrichments (gravel, shelters, changes in water flow direction), scaled to the number of fish in the tanks, following the procedure described in Räihä et al. (2019) [35]. The fish were attended to daily and fed with commercial fish feed.

### 4.1. Experimental Design

**Figure 2:**
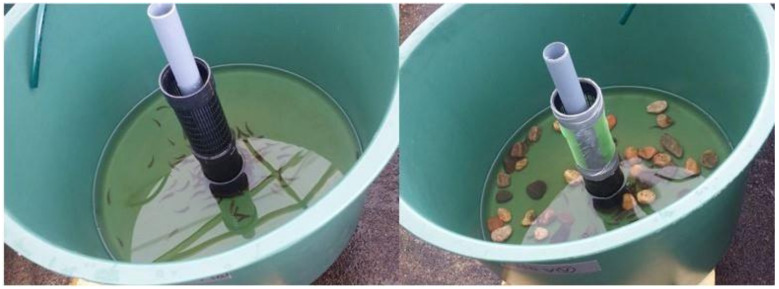
Standard and enriched tanks used in the experimental bacterial exposures. Replicated tanks with no stones (**left** panel), few stones (6 stones) or many stones (60 stones, **right** panel) were used. In addition, stones were either clean, unconditioned or conditioned in lake water before the experiment. Each tank (0.4 m^2^) had 100 fish (either trout or salmon) and 60 l of water (16.5–18.9 °C).

Fish of both species (mean length ± SE: 40.1 ± 0.09 mm (trout), 41.7 ± 0.08 mm (salmon)) were transferred to 50 tanks (0.4 m^2^) for the bacterial exposure, each tank with 60 L of water and 100 fish (either salmon or trout). Five different stone treatments were used for each species: tanks without stones, tanks with few stones (6 stones, 40–60 mm in diameter each) or with many stones (~60 stones of the same size, total volume 5 l, distributed randomly to the tank) that were either unconditioned or conditioned (Figure 2). Conditioning was done by keeping the stones in lake water (10–18 °C) in replicated rearing tanks for approximately seven weeks prior to the experiment. These tanks were separate from the housing tanks of the experimental fish (see above) to prevent possible familiarization effects of the fish to their home tanks. Unconditioned stones from the same lot had not been in the lake water before the experiment and were washed with tap water and sprayed with alcohol before the experiment. Each treatment combination had four replicate tanks (2 species × 5 treatments × 4 replicates = 40 tanks) and additionally one unexposed control tank for each combination (2 species × 5 treatments × 1 tank = 10 tanks). Stones and fish were placed into experimental tanks with constant water inflow 24 h before the exposure to allow the fish to recover from the transfer.

The *F. columnare* strain B351 used in the experimental exposures was originally isolated from the outlet water of a fish farm [62] and had been stored as pure culture at −80 °C in a stock containing 10% glycerol and 10% foetal calf serum. Before the experiment, the strain was revived overnight, inoculated in modified Shieh broth [63] and grown at 25.0 °C with constant agitation (100 rpm). After 23 h, the bacterial culture was enriched and used for infection. The exposure started when 150 mL of fresh, overnight-grown bacterial culture was added into each tank to reach the infection dose of 1.0 × 10^6^ colony-forming units per mL. In control tanks, 150 mL of Shieh broth without bacteria was added. The fish were exposed to the bacteria in 30 L of water for 1 h, after which the incoming water was turned on and the water level brought back to 60 L.

The condition of the fish was then monitored every six hours for the first 48 h and every four hours afterwards. Fatally moribund fish suffering from signs of flavobacterial infection (bleaching of the dorsal skin, loss of normal swimming buoyancy and mobility), were removed from the experiment at each inspection, euthanized using an overdose of benzocaine anaesthetic (200 mg/L), and measured for total length. All fish surviving the experiment (including control fish) were euthanized at the end of the experiment at 112 h post-exposure and measured for total length. Water temperature was 16.5–18.9 °C during the experiment.

To verify *F. columnare* infection, bacterial samples were taken from the first five moribund fish per tank. If a tank showed low fish mortality (e.g., the control tanks), five haphazardly chosen fish (*n* = 5) were sampled at the end of the experiment. Bacterial cultures were taken on Shieh agar plates supplemented with tobramycin (1 μg/mL, [64]). After the incubation of 48 h at room temperature, the appearance of yellow colonies with rhizoid morphology, typical for *F. columnare,* indicated positive bacterial infection. All exposed tanks had fish positive for *F. columnare*. Among the individual fish, 71.1% of the samples were positive (this was most likely due to the low air temperature at the time of bacterial sampling as similar exposures in laboratory conditions typically result in >95% prevalence [19]). All samples from fish deceased in the control tanks were negative for *F. columnare*. The study was conducted according to the guidelines of the Declaration of Helsinki and approved by the Regional State Administrative Agency of Southern Finland (ESAVI/8187/2018).

### 4.2. Statistical Analyses

Survival differences between the treatments were analysed using generalized linear mixed models (GLMM with binomial probability distribution) separately for each fish species. Stone quantity (few/many) and quality (unconditioned/conditioned) were used as fixed factors, the exposure tank nested under the quantity×quality interaction as a random factor and fish length as a covariate. Pairwise comparisons on the effects of individual factors, as well as comparisons between tanks with a few stones and those without stones used the same approach. All analyses were performed in R 3.3.2.

## Figures and Tables

**Table 1:** Survival is influenced by an interaction between the quantity and quality of enrichments. Here, the results of generalized linear mixed model (GLMM) analysis on the survival of trout exposed to *Flavobacterium columnare* in tanks with different quantity (few/many) and quality (unconditioned/conditioned) of stones are shown. The quantity and quality of the stones were used as fixed factors, the exposure tank as a random factor, and the fish length as a covariate.

Source	χ^2^	Degrees of Freedom	*p*
Quantity of stones	18.043	1	<0.001
Quality of stones	4.333	1	0.037
Quantity×Quality	4.933	1	0.026
Fish length	0.067	1	0.796

## Data Availability

Data are available in JYX Digital Repository of the University of Jyvaskyla: https://doi.org/10.17011/jyx/dataset/74704 (accessed on 15 December 2020).
